# First-line treatment with TKI plus brain radiotherapy versus TKI alone in EGFR-mutated non-small cell Lung cancer with brain metastases: a systematic review and meta-analysis

**DOI:** 10.1186/s12885-023-11548-0

**Published:** 2023-10-30

**Authors:** Yaowen Song, Shuiyu Lin, Jun Chen, Jun Dang

**Affiliations:** 1 Department of Radiation Oncology, The First Hospital of China Medical University, 155 Nanjing Road, Heping District, Shenyang, 110001 China; 2 Department of Radiation Oncology, Harbin Medical University Cancer Hospital, Harbin, China; 3 Department of Radiation Oncology, Shenyang Tenth People’s Hospital, Shenyang, China

**Keywords:** Non-small-cell Lung cancer, Brain metastases, Epidermal growth factor receptor tyrosine kinase inhibitors, Brain radiotherapy, meta-analysis

## Abstract

**Background:**

It remains uncertain whether first-line treatment with upfront brain radiotherapy (RT) in combined with epidermal growth factor receptor tyrosine kinase inhibitors (EGFR-TKIs) is superior to EGFR-TKIs alone for EGFR-mutated non-small cell lung cancer with newly diagnosed brain metastases (BMs). Therefore, we performed a meta-analysis to address this issue.

**Methods:**

We searched PubMed, Embase, Cochrane Library, and Web of Science databases for eligible studies published until February 28, 2023. The primary outcomes of interest were overall survival (OS) and intracranial progression-free survival (iPFS), reported as hazard ratios (HRs) and 95% confidence intervals (CIs).

**Results:**

Twenty-four retrospective studies with 3184 patients were included. First- or second-generation EGFR-TKIs were used in each study. Upfront brain RT plus EGFR-TKIs significantly prolonged OS (HR = 0.75, 95% CI: 0.64–0.88) and iPFS (HR = 0.61, 95% CI: 0.52–0.72) compared to EGFR-TKIs alone. There were no significant differences in OS and iPFS benefits from the combination therapy between asymptomatic and symptomatic patients, patients with exon 19 and 21 mutations, patients with 1–3 and > 3 BMs, and males and females, respectively (HRs interaction, P > 0.05 for each subgroup comparison).

**Conclusions:**

First-line treatment with upfront brain RT plus EGFR-TKIs is likely to be more effective than EGFR-TKIs alone. The benefits of combination therapy did not appear to be significantly affected by BM-related symptoms, EGFR mutation subtype, number of BMs, or sex.

**Supplementary Information:**

The online version contains supplementary material available at 10.1186/s12885-023-11548-0.

## Introduction

Non-small cell lung cancer (NSCLC) accounts for 85% of all lung cancers [1]. Epidermal growth factor receptor (EGFR) mutations are present in approximately 40% of Asian and 10–20% of non-Asian patients [2], with a 50–70% risk of developing brain metastases (BMs) [3]. Currently, EGFR tyrosine kinase inhibitors (EGFR-TKIs) are the standard first-line treatment for advanced NSCLC with EGFR mutations. However, first- and second-generation EGFR-TKIs have low cerebrospinal fluid (CSF) penetration rates (< 6%) [4]. Although third-generation osimertinib is better able to permeate the CSF than first- and second-generation EGFR-TKIs, its concentration in the CSF is far lower than that in the plasma [5, 6].

Brain radiotherapy (RT) has been shown to damage the blood-brain barrier (BBB) and increase the concentration of EGFR-TKIs in the CSF [7]. In addition, RT can reduce EGFR-TKIs resistance [8]. Therefore, EGFR-TKIs in combination with brain RT may be more effective than EGFR-TKIs alone theoretically. However, no randomized controlled trials (RCTs) have compared the two treatment strategies, and the results from retrospective studies are inconsistent [9–33]. It is possible that patients characteristics (such as EGFR mutation subtype, BM-related symptom, and number of BMs) or brain RT techniques affect the efficacy of the combination therapy. Although there were several published meta-analyses [34–37] of this subject have been published, the results had low statistical power as they were limited by the small number of included studies, which had small sample sizes, and few subgroup analyses.

In light of these issues, we performed a more comprehensive systematic review and meta-analysis of the currently available evidences, aiming to determine whether first-line treatment with upfront brain RT plus EGFR-TKIs was superior to EGFR-TKIs alone in patients with EGFR-mutated NSCLC with newly diagnosed BMs, and to explore the advantageous groups of the combination therapy by subgroup analyses.

## Materials and methods

This systematic review and meta-analysis was conducted in accordance with the Preferred Reporting Items for Systematic Reviews and Meta-analysis (PRISMA) guidelines [38], and was registered on the INPLASY international platform of registered systematic review and meta-analysis protocols (INPLASY202310013). The PRISMA checklist is shown in Table S1.

### Literature search

A systematic search of PubMed, Embase, Cochrane Library, and Web of Science before February 28, 2023 was performed independently by two authors (SY and LS). Search terms mainly included: (“non-small cell lung cancer” or “non-small cell lung carcinoma”); (“brain metastases” or “brain metastasis”); (“epidermal growth factor receptor” or “EGFR”); (“tyrosine kinase inhibitor” or “TKI”); “targeted therapy”; “gefitinib”; “erlotinib”; “icotinib”; “afatinib”; “dacomitinib”; “osimertinib”; and (“irradiation” or “radiation” or “radiotherapy”). The full set of search terms and detailed strategy of each database search are listed in Table S2. The references of the relevant reviews were manually checked to obtain additional articles.

### Inclusion and exclusion criteria

Studies were included if they met the following criteria: (1) study design: prospective or retrospective studies; (2) study population: histologically proven EGFR-mutated NSCLC, with newly diagnosed BMs identified by CT or MRI; (3) intervention: compared first-line treatment with upfront brain RT plus EGFR-TKIs with EGFR-TKIs alone; (4) outcomes: at least overall survival (OS) or intracranial progression-free survival (iPFS) reported; and (5) published in English. Upfront brain RT was defined as brain RT performed before the progression of intracranial disease to the first-line EGFR-TKIs therapy. Patients who received EGFR-TKIs prior to the diagnosis of BMs and those with ALK mutations were excluded.

### Data extraction and quality assessment

Two authors (SY and LS) independently extracted the patients baseline characteristics and data on OS, iPFS, intracranial objective response rate (iORR), and intracranial disease control rate (iDCR) from each study. OS was generally measured from the start of EGFR TKI therapy or the date of BM diagnosis until death or the last follow-up, whereas iPFS was calculated from the start of EGFR TKI therapy or the date of BM diagnosis until intracranial progression or the last follow-up. The iORR and iDCR were generally assessed by brain computed tomography (CT) or magnetic resonance imaging (MRI) using the Response Evaluation Criteria in Solid Tumors (RECIST, version 1.1) criteria. Responses were divided into complete remission (CR), partial remission (PR), stable disease (SD), and progressive disease (PD). iORR was calculated as CR + PR and iDCR was calculated as CR + PR + SD.

The Newcastle-Ottawa Scale (NOS) [39] was used to assess the quality of the retrospective studies. Grading of Recommendations Assessment, Development, and Evaluations (GRADE) was used to assess the quality of the evidence [40].

### Statistical analysis

Statistical analyses were performed using Review Manager Software version 5.3 (RevMan v5.3, Cochrane Collaboration, Oxford, UK). The outcomes of interest were OS, iPFS, iORR, and iDCR, presented as hazard ratios (HRs) or odds ratios (ORs) with their 95% confidence intervals (CIs). When not directly reported in the articles, HRs with 95% CIs were calculated using the Kaplan Meier curves [41, 42]. A random-effects model was used for the statistical analysis. The heterogeneity was assessed using the Chi-square (χ^2^) and I-square (I^2^) tests. Subgroup analyses of OS and iPFS were performed according to BMs related symptom (asymptomatic and symptomatic), EGFR mutation subtype (19 and 21 deletion mutations), number of BMs (1–3 and > 3), and sex (male and female). Sensitivity analysis was performed to evaluate the stability of the results. Publication bias was estimated using the funnel plots, Begg’s test [43], and Egger’s test [44].

## Results

### Literature search and study selection

**Fig. 1:**
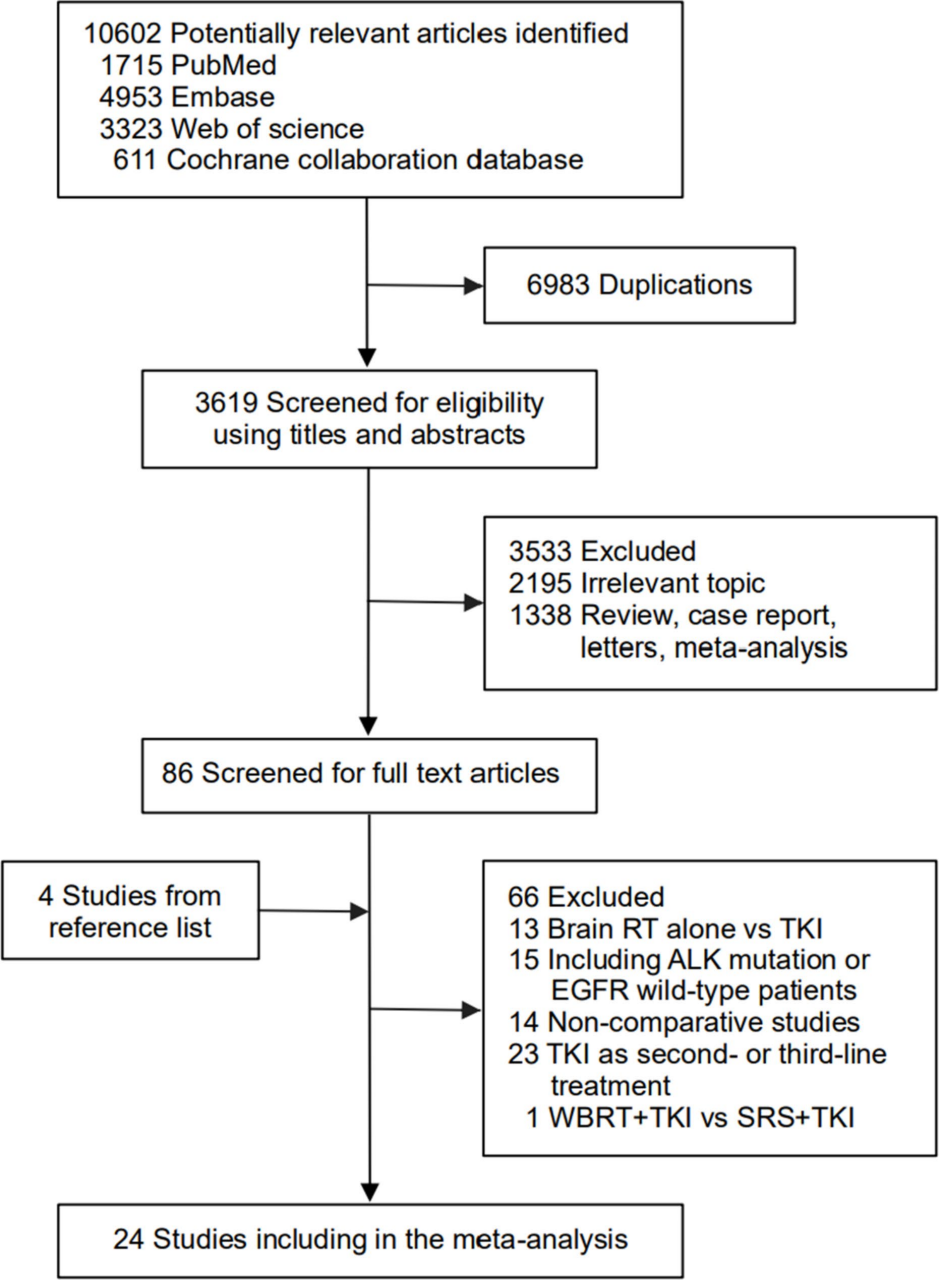
Literature search and selection

In total, 10,602 studies were included in the initial search. After removing the duplicates and screening the abstracts and/or full text, 10,581 studies were excluded. Finally, 24 retrospective studies [9–32] with 3184 patients were eligible for inclusion. The study selection process and reasons for exclusion are shown in Fig. 1. The majority of studies were conducted in Asia (23/24). First-generation (gefitinib, erlotinib, or icotinib) or second-generation (afatinib) EGFR-TKIs were used in all studies. The median follow up time was 22 months (interquartile range [IQR], 18–31). The median sample size was 55 participants (IQR, 38–66) in the upfront brain RT plus EGFR-TKIs group, and 60 participants (IQR, 40–88) in the EGFR-TKIs alone group. The percentage of males (34% vs. 32%), never smoking (48% vs. 51%), ECOG ≥ 2 (35% vs. 36%), exon 19 mutation (41% vs. 37%), and BM number ≤ 3 (17% vs. 22%) appeared to be similar between upfront brain RT plus EGFR-TKIs and EGFR-TKIs alone groups, while asymptomatic BMs (34% vs. 55%) appeared to be unbalanced. All the HRs used in our meta-analysis were derived without adjusting for the baseline characteristics of the two populations, except in one study [32]. OS and iPFS were calculated from the start of EGFR-TKI therapy in 9 and 14 studies, respectively; and were measured from the date of BM diagnosis in 14 and 9 studies, respectively. The patients characteristics are shown in Table 1, and the treatments and main outcomes are listed in Table 2.

**Table 1:** Patients characteristics of included studies

First Author/Year	Country	Intervention	Age (median)	Male (%)	ECOG 0–2 (%)	Never smoker(%)	Del-19 mutation(%)	BM number ≤ 3(%)	Asymptomatic BMs(%)	EGFR mutation	Sample size
Byeon/2016 [9]	Korea	WBRT/SRS + TKI	60	39	100	76	66	NR	52	19/21	59
		TKI	60	24	100	78	57	NR	80		62
Jiang/2016 [10]	China	WBRT + TKI	NR	NR	100	NR	NR	NR	NR	19/21	30
		TKI	NR	NR	100	NR	NR	NR	NR		91
Chen/2016 [11]	China	WBRT + TKI	52	45	100	68	53	21	30	19/21	53
		TKI	52	34	100	77	56	30	65		79
Zhu/2017 [12]	China	WBRT/SRS + TKI	NR	45	91	NR	50	27	NR	19/21	67
		TKI	NR	47	92	NR	42	35	NR		66
Liu/2017 [13]	China	WBRT/SRS + TKI	54	39	NR	76	41	25	20	19/21/others	49
		TKI	NR	31	NR	77	41	11	78		64
Fan/2017 [14]	China	WBRT/SRS + TKI	56	39	91	71	61	41	63	19/21	56
		TKI	59	51	83	63	56	66	88		41
Magnuson/2017 [15]	USA	WBRT/SRS + TKI	NR	34	NR	37	63	NR	55	19/20/21	220
		TKI	60	31	NR	32	62	NR	88		131
Wang/2018 [16]	China	WBRT/SRS + TKI	NR	NR	100	NR	NR	NR	NR	19/21/others	119
		TKI	NR	NR	100	NR	NR	NR	NR		62
Sung/2018 [17]	Korea	WBRT/SRS + TKI	NR	53	100	53	NR	NR	80	18/19/21	40
		TKI	NR	44	100	68	NR	NR	98		41
Li/2018 [18]	China	WBRT + TKI	57	41	82	77	47	NR	NR	19/21	17
		TKI	62	36	100	82	73	NR	NR		11
Chen/2018 [19]	China	WBRT + TKI	NR	44	100	45	56	14	24	19/21	66
		TKI	NR	36	100	49	56	18	23		39
Ke/2018 [20]	China	WBRT + TKI	53	47	100	67	53	23	NR	19/21	60
		TKI	52	34	100	77	56	30	NR		79
Yu/2018 [21]	China	WBRT/SRS + TKI	NR	NR	NR	NR	NR	NR	NR	19/21/others	24
		TKI	NR	NR	NR	NR	NR	NR	NR		18
Wang/2019 [22]	China	WBRT/SRS + TKI	NR	47	100	66	53	45	62	19/21/others	53
		TKI	NR	48	100	60	53	55	80		40
LEE/2019 [23]	China	WBRT/SRS + TKI	NR	36	NR	61	55	NR	NR	19/21	96
		TKI	NR	35	NR	68	42	NR	NR		102
Chen/2019 [24]	China	WBRT/SRS + TKI	59	41	NR	59	45	43	47	19/21/others	49
		TKI	59	45	NR	55	31	55	76		29
An/2019 [25]	China	WBRT/SRS + TKI	NR	40	100	77	46	60	NR	19/21	35
		TKI	NR	28	100	69	48	66	NR		29
Saida/2019 [26]	Japan	WBRT/SRS + TKI	71	36	82	56	38	56	49	18/19/21	39
		TKI	67	37	83	60	57	52	85		65
Saruwatari/2019 [27]	Japan	WBRT/SRS + TKI	NR	NR	NR	NR	NR	NR	NR	19/21	30
		TKI	NR	NR	NR	NR	NR	NR	NR		51
He/2019 [28]	China	WBRT + TKI	NR	56	100	41	46	47	48	19/21/others	56
		TKI	NR	44	100	47	54	53	52		48
Hyun/2020 [29]	Korea	WBRT/SRS + TKI	NR	41	NR	71	61	NR	65	18/19/21	66
		TKI	NR	35	NR	66	64	NR	92		107
Liu/2021 [30]	China	WBRT/SRS + TKI	NR	39	NR	71	48	36	49	19/21/others	77
		TKI	NR	33	NR	68	47	56	84		57
Gu/2021 [31]	China	WBRT/SRS + TKI	61	48	98	60	35	57	32	19/21/others	60
		TKI	62	43	96	63	37	61	67		186
Zhao/2022 [32]	China	WBRT + TKI	NR	NR	NR	NR	NR	NR	NR	19/21/others	94
		TKI	NR	NR	NR	NR	NR	NR	NR		171

Abbreviations: WBRT, whole-brain radiotherapy; SRS, stereotactic radiosurgery; TKI, epidermal growth factor receptor tyrosine kinase inhibitor; NR, not reported

**Table 2:** Treatments and main outcomes of included studies

First Author/Year	Intervention	RT dose/fraction	TKI drug	OS (HR, 95%CI)	iPFS (HR, 95%CI)	Follow up time(months)
Byeon/2016 [9]	WBRT/SRS + TKI	WBRT:20 Gy/10f; SRS:NR	Gefitinib/Erlotinib	1.16(0.50–2.73)	1.20(0.82–1.77)	18
	TKI					
Jiang/2016 [10]	WBRT + TKI	30 Gy/10f	Gefitinib/Erlotinib/Icotinib	1.67(1.00-3.12)	1.32(0.83–2.23)	NR
	TKI					
Chen/2016 [11]	WBRT + TKI	30 Gy/10f	Gefitinib/Erlotinib	1.03(0.59–1.80)	0.59(0.41–0.84)	36
	TKI					
Zhu/2017 [12]	WBRT/SRS + TKI	WBRT:30-40 Gy/10-20f; SRS:20 Gy/NR	Gefitinib/Erlotinib	0.55(0.34–0.90)	0.62(0.41–0.93)	18
	TKI					
Liu/2017 [13]	WBRT/SRS + TKI	WBRT:30-37.5y/10-15f; SRS:NR	Gefitinib/Erlotinib/Icotinib	0.33(0.12–0.87)	0.34(0.19–0.61)	30
	TKI					
Fan/2017 [14]	WBRT/SRS + TKI	WBRT:30 Gy/10f; SRS:20 Gy/NR	Icotinib	0.78(0.52–1.17)	0.66(0.44–0.99)	27
	TKI					
Magnuson/2017 [15]	WBRT/SRS + TKI	NR	Erlotinib	0.53(0.30–0.94)	0.82(0.65–1.04)	22
	TKI					
Wang/2018 [16]	WBRT/SRS + TKI	WBRT:30 Gy/10f; SRS:18.2 Gy/NR	Icotinib/Gefitinib/Erlotinib	0.74(0.55–0.99)	0.73(0.55–0.98)	17
	TKI					
Sung/2018 [17]	WBRT/SRS + TKI	NR	Gefitinib/Erlotinib	0.78(0.42–1.45)	0.39(0.21–0.72)	20
	TKI					
Li/2018 [18]	WBRT + TKI	NR	Afatinib	0.99(0.21–4.82)	NR	17
	TKI					
Chen/2018 [19]	WBRT + TKI	30 -40 Gy/10-20f	Gefitinib/Erlotinib/Icotinib	0.44(0.31–0.63)	0.18(0.07–0.43)	NR
	TKI					
Ke/2018 [20]	WBRT + TKI	30 Gy/10f	Gefitinib/Erlotinib	1.15(0.70–1.91)	0.54(0.37–0.78)	37
	TKI					
Yu/2018 [21]	WBRT/SRS + TKI	NR	Gefitinib/Erlotinib	NR	0.47(0.24–0.90)	NR
	TKI					
Wang/2019 [22]	WBRT/SRS + TKI	NR	Gefitinib/Erlotinib/Afatinib/ Icotinib	0.75(0.39–1.44)	0.38(0.19–0.75)	38
	TKI					
LEE/2019 [23]	WBRT/SRS + TKI	WBRT:30 Gy/10f; SRS:NR	Gefitinib/Erlotinib/Afatinib	0.52(0.20–1.32)	NR	NR
	TKI					
Chen/2019 [24]	WBRT/SRS + TKI	WBRT:30 Gy/10f; SRS:25-35 Gy/5f	Gefitinib/Erlotinib/Icotinib	0.51(0.81–1.28)	0.33(0.16–0.69)	NR
	TKI					
An/2019 [25]	WBRT/SRS + TKI	WBRT:30 Gy/10f; SRS:30-45 Gy/5-10f	Gefitinib/Erlotinib/Icotinib	0.40(0.17–0.93)	0.43(0.21–0.91)	31
	TKI					
Saida/2019 [26]	WBRT/SRS + TKI	WBRT:30 Gy/10f; SRS:20-30 Gy/1-4f	Gefitinib/Erlotinib/Afatinib	0.86(0.53–1.36)	0.82(0.53–1.27)	22
	TKI					
Saruwatari/2019 [27]	WBRT/SRS + TKI	NR	Gefitinib/Erlotinib	0.42(0.20–0.88)	NR	18
	TKI					
He/2019 [28]	WBRT + TKI	30 Gy/10f	Gefitinib/Erlotinib/Icotinib	0.83(0.54–1.27)	0.58(0.37–0.89)	23
	TKI					
Hyun/2020 [29]	WBRT/SRS + TKI	NR	Gefitinib/Erlotinib/Afatinib	1.34(0.99–1.83)	0.72(0.42–1.23)	19
	TKI					
Liu/2021 [30]	WBRT/SRS + TKI	Mixed	Gefitinib/Erlotinib/Icotinib	0.56(0.34–0.94)	0.51(0.33–0.79)	28
	TKI					
Gu/2021 [31]	WBRT/SRS + TKI	NR	Gefitinib/Erlotinib/Icotinib/Afatinib/Dacomitinib/ Osimertinib/Almonertinib	0.92(0.72–1.18)	0.75(0.58–0.96)	40
	TKI					
Zhao/2022 [32]	WBRT + TKI	30 Gy/10f	Gefitinib/Erlotinib/ Osimertinib	0.66(0.47–0.91)	NR	17
	TKI					

Abbreviations: OS, overall survival; iPFS, intracranial progression-free survival; WBRT, whole-brain radiotherapy; SRS, stereotactic radiosurgery; TKI, epidermal growth factor receptor tyrosine kinase inhibitor; NR, not reported

### Assessment of study and evidence and publication bias

All studies were judged with a score of ≥ 6 (Table S3). The GRADE assessment results for each finding are shown in Table S4. The evidence for OS, iORR, and iDCR had moderate GRADE ratings, while the evidence for iPFS had very low GRADE ratings. The evidence for all the outcomes of subgroup analyses of OS were of low and very low GRADE. Regarding the evidences for the outcomes of subgroup analyses of iPFS, asymptomatic BMs, BM number > 3, and female sex had moderate GRADE rating, and the evidence for the remaining outcomes was low or very low GRADE.

The Begg’s and Egger’s test results indicated a significant publication bias in iPFS (Begg’s test, P = 0.002; Egger’s test, P = 0.003), but not in OS (Begg’s test, P = 0.54; Egger’s test, P = 0.91). Funnel plots are shown in Fig S1.

### Upfront brain RT plus EGFR-TKIs vs. EGFR-TKIs alone

**Fig. 2:**
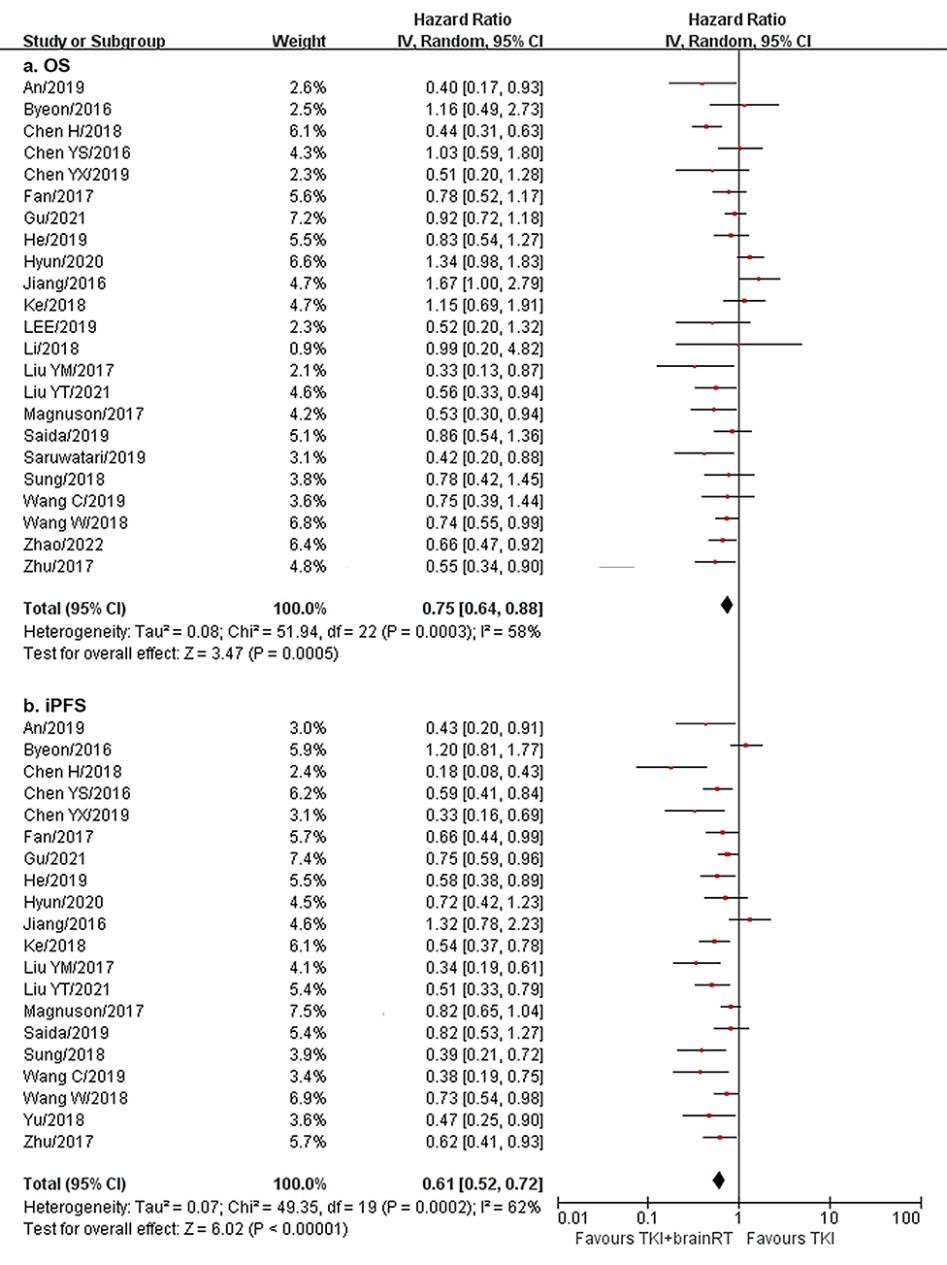
OS and iPFS of upfront brain RT plus EGFR-TKIs vs. EGFR-TKIs alone. OS, overall survival; iPFS, intracranial progression-free survival; RT, radiotherapy; EGFR-TKIs, epidermal growth factor receptor tyrosine kinase inhibitors

There were 23 studies with 3142 patients for OS and 20 studies with 2612 patients for iPFS. Upfront brain RT plus EGFR-TKIs showed significantly longer OS (HR = 0.75, 95% CI: 0.64–0.88, I^2^ = 58%) and iPFS (HR = 0.61, 95% CI: 0.52–0.72, I^2^ = 62%) compared to EGFR-TKIs alone (Fig. 2).

**Fig. 3:**
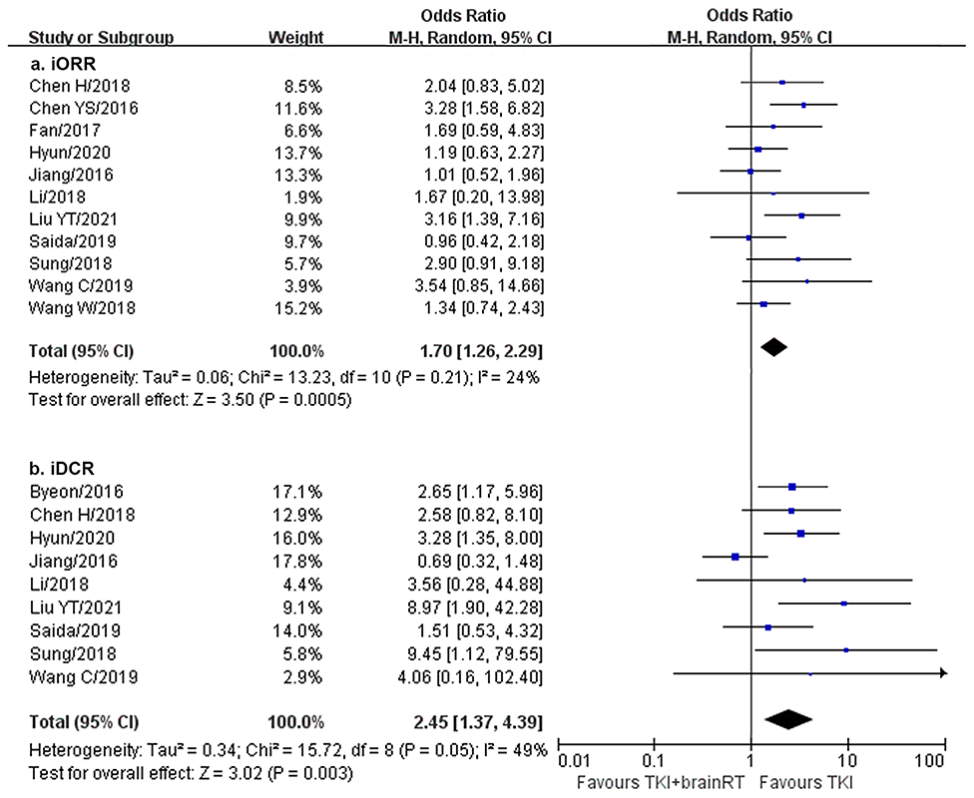
iORR and iDCR of upfront brain RT plus EGFR-TKIs vs. EGFR-TKIs alone. iORR, intracranial objective response rate; iDCR, intracranial disease control rate

There were 11 studies with 1295 patients for iORR and 9 studies with 1006 patients for iDCR. Compared to EGFR-TKIs alone, upfront brain RT plus EGFR-TKIs achieved significantly higher iORR (OR = 1.70, 95% CI: 1.26–2.29, I^2^ = 24%) and iDCR (OR = 2.45, 95% CI: 1.37–4.39, I^2^ = 49%) (Fig. 3).

### Subgroup analysis for upfront brain RT plus EGFR-TKIs vs. EGFR-TKIs alone

**Fig. 4:**
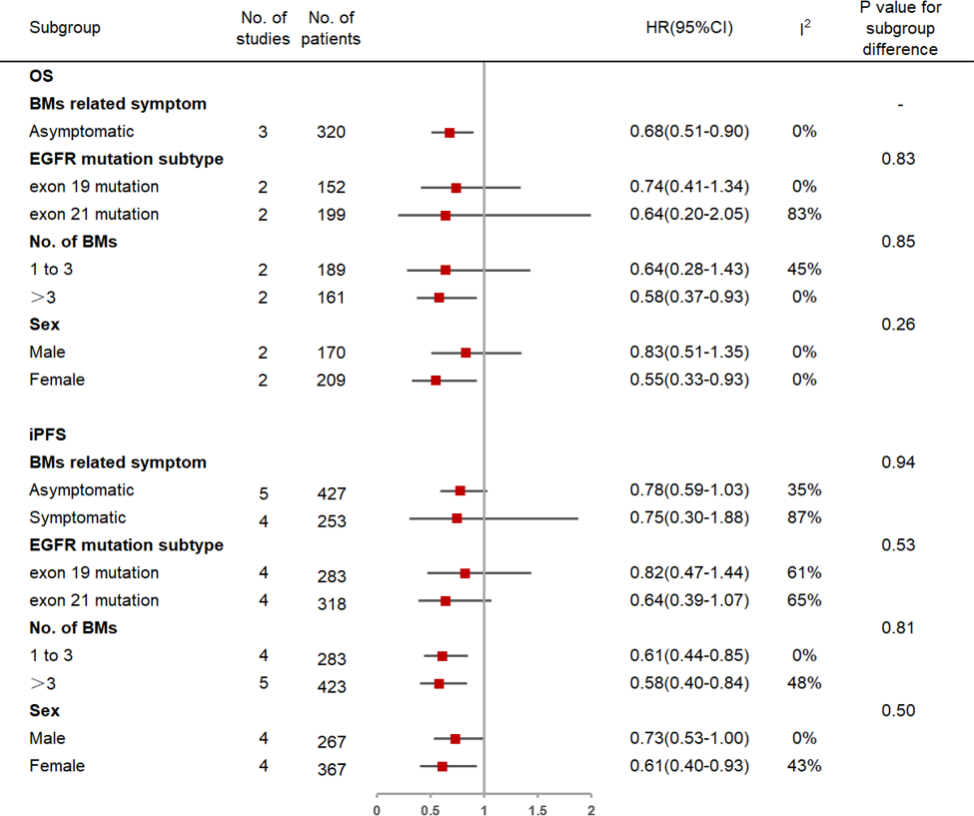
Subgroup analysis of upfront brain RT plus EGFR-TKIs vs. EGFR-TKIs alone. OS, overall survival; iPFS, intracranial progression-free survival; RT, radiotherapy; EGFR-TKIs, epidermal growth factor receptor tyrosine kinase inhibitors; BMs, brain metastases

The OS and iPFS results for each subgroup are shown in Fig. 4.

#### BM-related symptoms

The addition of upfront brain RT to EGFR-TKIs significantly prolonged OS (3 studies with 320 patients; HR = 0.68, 95% CI: 0.51–0.90, I^2^ = 0%) but iPFS (5 studies with 427 patients; HR = 0.78, 95% CI: 0.59–1.03, I^2^ = 35%) in patients with asymptomatic BMs. For symptomatic patients, no significant difference in iPFS was observed between EGFR-TKIs with and without upfront brain RT (4 studies with 253 patients; HR = 0.75, 95% CI: 0.30–1.88, I^2^ = 87%). There was no significant difference in iPFS benefit between asymptomatic and symptomatic patients (P_interaction_ = 0.94).

#### EGFR mutation subtype

There were no significant differences in OS and iPFS between upfront brain RT plus EGFR-TKIs and EGFR-TKIs alone either in the exon 19 mutation group (2 studies with 152 patients; HR = 0.74, 95% CI: 0.41–1.34, I^2^ = 0% and 4 studies with 283 patients; HR = 0.82, 95% CI: 0.47–1.44, I^2^ = 61%) or in the exon 21 mutation group (2 studies with 199 patients; HR = 0.64, 95% CI: 0.20–2.05, I^2^ = 83% and 4 studies with 318 patients; HR = 0.64, 95% CI: 0.39–1.07, I^2^ = 65%). P-values for subgroup differences in OS and iPFS benefits were 0.83 and 0.53, respectively.

#### Number of BMs

Upfront brain RT plus EGFR-TKIs significantly improved OS (2 studies with 161 patients; HR = 0.58, 95% CI: 0.37–0.93, I^2^ = 0%) and iPFS (5 studies with 423 patients; HR = 0.58, 95% CI: 0.40–0.84, I^2^ = 48%) compared to EGFR-TKIs alone in patients with > 3 BMs. As for patients with 1–3 BMs, the combination therapy significantly prolonged iPFS (4 studies with 283 patients; HR = 0.61, 95% CI: 0.44–0.85, I^2^ = 0%) but OS (2 studies with 189 patients; HR = 0.64, 95% CI: 0.28–1.43, I^2^ = 45%). No significant differences in OS (P_interaction_ = 0.85) or iPFS (P_interaction_ = 0.81) benefits were observed between the two subgroups.

#### Sex

Upfront brain RT plus EGFR-TKIs was associated with significantly longer OS and iPFS compared to EGFR-TKIs alone in females (2 studies with 209 patients; HR = 0.55, 95% CI: 0.33–0.93, I^2^ = 0% and 4 studies with 367 patients; HR = 0.61, 95% CI: 0.40–0.93, I^2^ = 43%, respectively), but not in males (2 studies with 170 patients; HR = 0.83, 95% CI: 0.51–1.35, I^2^ = 0% and 4 studies with 267 patients; HR = 0.73, 95% CI: 0.53-1.00, I^2^ = 0%, respectively). However, there were no significant differences in OS (P_interaction_ = 0.26) and iPFS (P_interaction_ = 0.50) benefits between the two sexes.

### Sensitivity analysis

When each study was omitted individually, the pooled HRs of OS or iPFS did not change markedly, suggesting a relatively stable result (Fig S2).

## Discussion

This comprehensive systematic review and meta-analysis assessed the efficacy of first-line treatment with upfront brain RT plus EGFR-TKIs versus EGFR-TKIs alone in patients with EGFR-mutated NSCLC with BMs. Compared to EGFR-TKIs alone, upfront brain RT plus EGFR-TKIs achieved significantly longer OS and iPFS, and higher iORR and iDCR. Nevertheless, the current results are based on retrospective studies with significant heterogeneity, and therefore, need to be validated in large RCTs. In addition, there are still challenges for the use of upfront brain RT, such as RT techniques and advantageous groups.

Historically, whole-brain radiotherapy (WBRT) has been the mainstay of local treatment modality for BMs. However, there is a growing concern regarding its neurological toxicity. Stereotactic radiosurgery (SRS) is known to have less neurotoxicity, and has now become a more widely used brain RT technique. However, whether SRS is superior to WBRT when combined with EGFR-TKIs remains unclear. In a retrospective study of patients with NSCLC with ≤ 3 BMs and EGFR-sensitive mutation [33], SRS + EGFR-TKIs was associated with significantly longer median OS compared to WBRT + EGFR-TKIs. In another retrospective study assessing the optimal treatment strategy for EGFR-mutant NSCLC with BMs [45], EGFR-TKIs plus SRS significantly improved OS compared to EGFR-TKI without SRS for patients with Lung-mol graded prognostic assessment (GPA) ≥ 3 but not for those with Lung-mol GPA < 3. In addition, no significant difference in OS was observed between EGFR-TKI with and without WBRT. These results suggest that first-line SRS plus EGFR-TKIs is more effective than WBRT plus EGFR-TKIs. Nevertheless, SRS is likely limited by the number of intracranial lesions [33] or Lung-mol GPA score [45]. For patients with more BM lesions or low Lung-mol GPA scores, the superiority of SRS requires further evaluation.

In terms of the number of BMs, Zhu et al. [12] and He et al. [28] found that upfront brain RT prolonged iPFS in patients with > 3 BMs, but not in patients with 1–3 BMs. However, Liu et al. [30] reported the better iPFS with upfront brain RT regardless of the number of BMs. In our study, although the upfront brain significantly improved iPFS both in the 1–3 and > 3 BMs groups, only patients with > 3 BMs had significantly longer OS. Nevertheless, no differences in OS (HR = 0.55 vs. 0.83, P = 0.26) and iPFS (HR = 0.58 vs. 0.73, P = 0.31) benefits were observed between the two groups. In addition, many patients with 1–3 BMs in brain RT group received WBRT in this study. As mentioned previously, SRS appears to be more effective than WBRT for patients with limited BM lesions. Thus, the number of BMs is unlikely to be an independent factor associated with the efficacy of upfront brain RT.

The necessity of upfront brain RT in patients with asymptomatic BM remains controversial. The current National Comprehensive Cancer Network (NCCN) guidelines for central nervous system cancers (Version 2.2022) recommend that first-line EGFR-TKIs alone should be considered in asymptomatic patients [46]. However, although EGFR-TKIs alone can prevent brain RT toxicities, they still pose a higher risk of subsequent intracranial progression. In our meta-analysis, upfront brain RT plus EGFR-TKIs significantly improved OS compared with EGFR-TKIs alone in patients with asymptomatic BMs. In addition, iPFS benefit was not significantly different between asymptomatic and symptomatic patients (HRs: 0.78 vs. 0.75; P = 0.94). These findings highlight the value of upfront brain RT for patients with asymptomatic BMs and suggest that first-line treatment with EGFR-TKIs monotherapy may be insufficient for this patients population.

EGFR-TKIs have demonstrated superior OS and PFS in patients with exon 19-deletion mutated NSCLC than compared to those with exon 21-deletion mutated NSCLC [47]. However, compared to low plasma concentration, a high plasma concentration of gefitinib was found to be associated with longer PFS in patients harboring exon 21 mutations but not in those harboring exon 19 mutations [48]. Combined brain RT can disrupt the BBB, leading to increased concentration of EGFR-TKIs in the CSF. Thus, patients with exon 21 mutations may benefit more from the addition of brain RT to EGFR-TKIs than patients with exon 19 mutations. However, we did not find significant difference in OS and iPFS benefits between the two mutation subtypes in this meta-analysis (HRs of OS: 0.74 vs. 0.64, P = 0.83; HRs of iPFS: 0.82 vs. 0.64, P = 0.53). Mutation subtype differences in the benefits of the combination therapy require further investigation in future RCTs.

Sex differences in the efficacy of EGFR-TKIs for NSCLC have also been investigated in clinical studies. A more recent review [49] summarized that women tended to benefit more from first-generation EGFR-TKIs than men in terms of PFS. However, whether sex affects the efficacy of the combination of EGFR-TKIs and brain RT in patients with NSCLC with BMs remains unclear. In our study, although upfront brain RT plus EGFR-TKIs was associated with significantly improved OS and iPFS in females but not in males, the difference in either OS or iPFS benefit between the two sexes did not reach statistical significance (HR of OS: 0.55 vs. 0.83, P = 0.26; HR of iPFS: 0.61 vs. 0.73, P = 0.31). Thus, it remains difficult to conclude whether females have a better response to upfront brain RT than males.

It should be noted that the results of this meta-analysis were based on the first- and second-generation TKIs. Third-generation osimertinib has demonstrated a better BBB penetration [5, 6], and higher iORR and iPFS [50] than first- and second-generation TKIs. These findings raise the question of whether upfront brain RT can be omitted in patients treated with first-line osimertinib therapy? In contrast, osimertinib has been reported to enhance radiosensitivity by reducing NSCLC cell cycle arrest in the G2/M phase, and blocking the repair of RT-induced DNA double-strand breaks [51], suggesting greater efficacy of osimertinib combined with RT. Currently, only a few clinical studies have been conducted on this topic. In a retrospective study by Zhao et al. [32], upfront SRS and/or surgery plus osimertinib was associated with improved OS compared with osimertinib alone. However, the addition of brain RT to osimertinib treatment failed to prolong PFS and OS in another retrospective study [52]. In addition, Cheng et al. [45] found that patients receiving first-line first- or secondgeneration EGFR-TKIs with sequential osimertinib had significantly longer survival than those without sequential osimertinib, regardless of the use of brain RT. Collectively, the value of upfront brain RT in the era of first-line osimertinib treatment requires further exploration.

Compared with the previous meta-analyses [34–37], our study included more studies with larger sample sizes. In addition, subgroup analyses of OS and iPFS were performed to identify advantageous groups. Moreover, our meta-analysis revealed some new findings, including that patients with asymptomatic BMs could also benefit from the upfront brain RT; and the benefits from the combination therapy did not appear to be influenced by BM-related symptoms, mutation subtype, number of BMs, or sex. These findings will be helpful in determining first-line treatment strategies for patients with NSCLC with EGFR mutations and newly diagnosed BMs.

Nevertheless, our meta-analysis had some limitations. First, all data were extracted from retrospective studies, which might have selection bias. In addition, clinical characteristics were unbalanced between groups in some studies. Moreover, the number of studies included in subgroup analyses was relatively small. All these factors may have resulted in lower reliability of our results. Second, there was significant heterogeneity and publication bias for OS and/or PFS. The results of the subgroup analyses indicated that BMs related symptoms, EGFR mutation subtype, number of BMs, and sex might be associated with the heterogeneity. In addition, the patients’ ECOG performance status and extracranial metastases were inconsistent among studies, which might also account for some heterogeneity. Third, most of the included studies were conducted in Asia, and generalizing the findings to other regions should be done with caution. Fourth, OS and iPFS were measured from the start of EGFR-TKI therapy or from the date of BM diagnosis. The inconsistent starting time points for OS and iPFS used in the included studies might have resulted in an immortal time bias. Fifth, some HRs were not directly reported in the texts, and were calculated using the Kaplan-Meier curves. This may also result in bias. Finally, treatment-related adverse events were not assessed in our study because of a lack of data.

## Conclusions

First-line treatment with upfront brain RT and EGFR-TKIs is likely to be more effective than EGFR-TKIs alone. The benefits of the combination therapy did not appear to be significantly affected by BM-related symptoms, EGFR mutation subtype, number of BMs, or sex. Nevertheless, these findings need to be confirmed in future RCTs. Whether these results can be extrapolated to third-generation EGFR-TKIs requires further investigation.

### Electronic supplementary material

Below is the link to the electronic supplementary material.

**Table S1** PRISMA Checklist. **Table S2** Search strategy. **Table S3** Quality assessment of retrospective studies using the Newcastle-Ottawa scale. **Table S4** GRADE assessment. **Fig S1** Funnel plots of publication bias. OS, overall survival; iPFS, intracranial progression-free survival. **Fig S2** Sensitivity analysis. OS, overall survival; iPFS, intracranial progression-free survival.

## Data Availability

All datasets generated for this study are included in the article/Supplementary Material. Further inquiries can be directed to the corresponding author.

## Abbreviations

RT: Radiotherapy
EGFR-TKIs: Epidermal growth factor receptor tyrosine kinase inhibitors
BMs: Brain metastases
OS: Overall survival
iPFS: Intracranial progression-free survival
HRs: Hazard ratios
CIs: Confidence intervals
WBRT: Whole-brain radiotherapy
SRS: Stereotactic radiosurgery
NSCLC: Non-small cell lung cancer
EGFR: Epidermal growth factor receptor
CSF: Cerebrospinal fluid
BBB: Blood-brain barrier
RCTs: Randomized controlled trials
PRISMA: Preferred reporting items for systematic reviews and meta-analysis
iORR: Intracranial objective response rate
iDCR: Intracranial disease control rate
NOS: Newcastle-ottawa scale
ORs: Odds ratios
IQR: Interquartile range
