# Supplementary material for: First-line treatment with TKI plus brain radiotherapy versus TKI alone in EGFR-mutated non-small cell Lung cancer with brain metastases: a systematic review and meta-analysis
Source: BMC Cancer. 2023 Oct 30;23:1043. doi: 10.1186/s12885-023-11548-0 (PMC10614414; doi:10.1186/s12885-023-11548-0)
Supplement: Supplementary file 1 — Table S1 PRISMA Checklist. Table S2 Search strategy. Table S3 Quality assessment of retrospective studies using the Newcastle-Ottawa scale. Table S4 GRADE assessment. Fig S1 Funnel plots of publication bias. OS, overall survival; iPFS, intracranial progression-free survival. Fig S2 Sensitivity analysis. OS, overall survival; iPFS, intracranial progression-free survival. [file 12885_2023_11548_MOESM1_ESM.doc]

**Table S1 PRISMA Checklist**

| **Section/Topic** | **Item #** | **Checklist Item** | **Reported on Page #** |
| --- | --- | --- | --- |
| **TITLE** |  |  |  |
| Title | 1 | Identify the report as a systematic review incorporating a meta-analysis (or related form of meta-analysis). | 1 |
| **ABSTRACT** |  |  |  |
| Structured summary | 2 | Provide a structured summary including, as applicable:  **Background:** main objectives  **Methods:** data sources; study eligibility criteria, participants, and interventions; study appraisal; and synthesis methods, such as network meta-analysis.  **Results:** number of studies and participants identified; summary estimates with corresponding confidence/credible intervals; treatment rankings may also be discussed. Authors may choose to summarize pairwise comparisons against a chosen treatment included in their analyses for brevity.  **Discussion/Conclusions:** limitations; conclusions and implications of findings.  **Other:** primary source of funding; systematic review registration number with registry name. | 2-3 |
| **INTRODUCTION** |  |  |  |
| Rationale | 3 | Describe the rationale for the review in the context of what is already known, including mention of why a network meta-analysis has been conducted. | 4-5 |
| Objectives | 4 | Provide an explicit statement of questions being addressed, with reference to participants, interventions, comparisons, outcomes, and study design (PICOS). | 5 |
| **METHODS** |  |  |  |
| Protocol and registration | 5 | Indicate whether a review protocol exists and if and where it can be accessed (e.g., Web address); and, if available, provide registration information, including registration number. | INPLASY202310013 |
| Eligibility criteria | 6 | Specify study characteristics (e.g., PICOS, length of follow-up) and report characteristics (e.g., years considered, language, publication status) used as criteria for eligibility, giving rationale. Clearly describe eligible treatments included in the treatment network, and note whether any have been clustered or merged into the same node (with justification). | 6 |
| Information sources | 7 | Describe all information sources (e.g., databases with dates of coverage, contact with study authors to identify additional studies) in the search and date last searched. | 5 |
| Search | 8 | Present full electronic search strategy for at least one database, including any limits used, such that it could be repeated. | Table S2 |
| Study selection | 9 | State the process for selecting studies (i.e., screening, eligibility, included in systematic review, and, if applicable, included in the meta-analysis). | 5 |
| Data collection process | 10 | Describe method of data extraction from reports (e.g., piloted forms, independently, in duplicate) and any processes for obtaining and confirming data from investigators. | 6 |
| Data items | 11 | List and define all variables for which data were sought (e.g., PICOS, funding sources) and any assumptions and simplifications made. | 6 |
| **Geometry of the network** | **S1** | Describe methods used to explore the geometry of the treatment network under study and potential biases related to it. This should include how the evidence base has been graphically summarized for presentation, and what characteristics were compiled and used to describe the evidence base to readers. | NA |
| Risk of bias within individual studies | 12 | Describe methods used for assessing risk of bias of individual studies (including specification of whether this was done at the study or outcome level), and how this information is to be used in any data synthesis. | 7 |
| Summary measures | 13 | State the principal summary measures (e.g., risk ratio, difference in means). Also describe the use of additional summary measures assessed, such as treatment rankings and surface under the cumulative ranking curve (SUCRA) values, as well as modified approaches used to present summary findings from meta-analyses. | 6-7 |
| Planned methods of analysis | 14 | Describe the methods of handling data and combining results of studies for each network meta-analysis. This should include, but not be limited to:   - Handling of multi-arm trials; - Selection of variance structure; - Selection of prior distributions in Bayesian analyses; and - Assessment of model fit. | 6-7 |
| **Assessment of Inconsistency** | **S2** | Describe the statistical methods used to evaluate the agreement of direct and indirect evidence in the treatment network(s) studied. Describe efforts taken to address its presence when found. | NA |
| Risk of bias across studies | 15 | Specify any assessment of risk of bias that may affect the cumulative evidence (e.g., publication bias, selective reporting within studies). | 7 |
| Additional analyses | 16 | Describe methods of additional analyses if done, indicating which were pre-specified. This may include, but not be limited to, the following:   - Sensitivity or subgroup analyses; - Meta-regression analyses; - Alternative formulations of the treatment network; and - Use of alternative prior distributions for Bayesian analyses (if applicable). | 7 |
| **RESULTS†** |  |  |  |
| Study selection | 17 | Give numbers of studies screened, assessed for eligibility, and included in the review, with reasons for exclusions at each stage, ideally with a flow diagram. | 8 |
| **Presentation of network structure** | **S3** | Provide a network graph of the included studies to enable visualization of the geometry of the treatment network. | NA |
| **Summary of network geometry** | **S4** | Provide a brief overview of characteristics of the treatment network. This may include commentary on the abundance of trials and randomized patients for the different interventions and pairwise comparisons in the network, gaps of evidence in the treatment network, and potential biases reflected by the network structure. | NA |
| Study characteristics | 18 | For each study, present characteristics for which data were extracted (e.g., study size, PICOS, follow-up period) and provide the citations. | Table 1,2 |
| Risk of bias within studies | 19 | Present data on risk of bias of each study and, if available, any outcome level assessment. | Table S3 |
| Results of individual studies | 20 | For all outcomes considered (benefits or harms), present, for each study: 1) simple summary data for each intervention group, and 2) effect estimates and confidence intervals. Modified approaches may be needed to deal with information from larger networks. | 9-11 |
| Synthesis of results | 21 | Present results of each meta-analysis done, including confidence/credible intervals. In larger networks, authors may focus on comparisons versus a particular comparator (e.g. placebo or standard care), with full findings presented in an appendix. League tables and forest plots may be considered to summarize pairwise comparisons. If additional summary measures were explored (such as treatment rankings), these should also be presented. | 9-11 |
| **Exploration for inconsistency** | **S5** | Describe results from investigations of inconsistency. This may include such information as measures of model fit to compare consistency and inconsistency models, P values from statistical tests, or summary of inconsistency estimates from different parts of the treatment network. | NA |
| Risk of bias across studies | 22 | Present results of any assessment of risk of bias across studies for the evidence base being studied. | 9 |
| Results of additional analyses | 23 | Give results of additional analyses, if done (e.g., sensitivity or subgroup analyses, meta-regression analyses, alternative network geometries studied, alternative choice of prior distributions for Bayesian analyses, and so forth). | 10-11 |
| **DISCUSSION** |  |  |  |
| Summary of evidence | 24 | Summarize the main findings, including the strength of evidence for each main outcome; consider their relevance to key groups (e.g., healthcare providers, users, and policy-makers). | 11-16 |
| Limitations | 25 | Discuss limitations at study and outcome level (e.g., risk of bias), and at review level (e.g., incomplete retrieval of identified research, reporting bias). Comment on the validity of the assumptions, such as transitivity and consistency. Comment on any concerns regarding network geometry (e.g., avoidance of certain comparisons). | 16-17 |
| Conclusions | 26 | Provide a general interpretation of the results in the context of other evidence, and implications for future research. | 17 |
|  |  |  |  |
| **FUNDING** |  |  |  |
| Funding | 27 | Describe sources of funding for the systematic review and other support (e.g., supply of data); role of funders for the systematic review. This should also include information regarding whether funding has been received from manufacturers of treatments in the network and/or whether some of the authors are content experts with professional conflicts of interest that could affect use of treatments in the network. | NA |

PICOS = population, intervention, comparators, outcomes, study design.

**Table S2** Search strategy

**a:** Search strategy in PubMed

| # | Query |
| --- | --- |
| #1 | “Carcinoma, Non-Small-Cell Lung”[mh] |
| #2 | Non Small Cell Lung Carcinoma[tiab] OR Non-Small Cell Lung Carcinoma[tiab] OR Non-Small-Cell Lung Carcinoma[tiab] OR Carcinoma, Non Small Cell Lung[tiab] OR Carcinoma, Non-Small Cell Lung[tiab] OR Lung Carcinoma, Non-Small-Cell[tiab] OR Non Small Cell Cancer Of The Lung[tiab] OR Non Small Cell Lung Cancer[tiab] OR NSCLC[tiab] |
| #3 | #1 OR #2 |
| #4 | Brain Metastases[tiab] OR Brain Metastase[tiab] OR Brain Metastasis[tiab] |
| #5 | “Radiotherapy”[mh] OR Radiotherapies[tiab] OR Radiation Therapy[tiab] OR Radiation Therapies[tiab] OR Therapy, Radiation[tiab] OR Therapies, Radiation[tiab] OR Radiation Treatment[tiab] OR Treatment, Radiation[tiab] OR Targeted Radiotherapy[tiab] OR Radiotherapy, Targeted[tiab] OR Targeted Radiation Therapy[tiab] OR Therapy, Targeted Radiation[tiab] OR Radiation Therapy, Targeted[tiab] |
| #6 | Epidermal Growth Factor Receptor[tiab] OR EGFR[tiab] OR Receptor, Epidermal Growth Factor[tiab] OR Tyrosine Kinase Inhibitor[tiab] OR TKI[tiab] OR Inhibitors, Tyrosine Kinase[tiab] OR Targeted Therapy[tiab] OR Gefitinib[tiab] OR Erlotinib[tiab] OR Icotinib[tiab] OR Afatinib[tiab] OR Dacomitinib[tiab] OR Osimertinib[tiab] |
| #7 | #5 OR #6 |
| #8 | #3 AND #4 AND #7 |

**b: Search strategy in Embase**

| # | Query |
| --- | --- |
| #1 | 'Carcinoma, Non-Small-Cell Lung'/exp |
| #2 | 'Carcinoma, Non-Small-Cell Lung':ab,ti OR 'NSCLC':ab,ti |
| #3 | #1 OR #2 |
| #4 | 'Brain Metastases':ab,ti OR 'Brain Metastase':ab,ti OR 'Brain Metastasis':ab,ti |
| #5 | 'Radiotherapy'/exp OR 'Radiotherapies':ab,ti OR 'Radiation Therapy':ab,ti OR 'Radiation Therapies':ab,ti OR 'Therapy, Radiation':ab,ti OR 'Therapies, Radiation':ab,ti OR 'Radiation Treatment':ab,ti OR 'Treatment, Radiation':ab,ti OR 'Targeted Radiotherapy':ab,ti OR 'Radiotherapy, Targeted':ab,ti OR 'Targeted Radiation Therapy':ab,ti OR 'Therapy, Targeted Radiation':ab,ti OR 'Radiation Therapy, Targeted':ab,ti |
| #6 | 'Epidermal Growth Factor Receptor':ab,ti OR 'EGFR':ab,ti OR 'Receptor, Epidermal Growth Factor':ab,ti OR 'Tyrosine Kinase Inhibitor':ab,ti OR 'TKI':ab,ti OR 'Inhibitors, Tyrosine Kinase':ab,ti OR 'Targeted Therapy':ab,ti OR 'Gefitinib':ab,ti OR 'Erlotinib':ab,ti OR 'Icotinib':ab,ti OR 'Afatinib':ab,ti OR 'Dacomitinib':ab,ti OR 'Osimertinib':ab,ti |
| #7 | #5 OR #6 |
| #8 | #3 AND #4 AND #7 |

**c:** Search strategy in Cochrane Library

| # | Query |
| --- | --- |
| #1 | MeSH descriptor: [Carcinoma, Non-Small-Cell Lung] explode all trees |
| #2 | (Non Small Cell Lung Cancer) OR (NSCLC) |
| #3 | #1 OR #2 |
| #4 | (Brain Metastases OR Brain Metastase OR Brain Metastasis):ti,ab |
| #5 | (Radiotherapy OR Radiotherapies OR Radiation Therapy OR Radiation Therapies OR Therapy, Radiation OR Therapies, Radiation OR Radiation Treatment OR Treatment, Radiation OR Targeted Radiotherapy OR Radiotherapy, Targeted OR Targeted Radiation Therapy OR Therapy, Targeted Radiation OR Radiation Therapy, Targeted):ti,ab |
| #6 | (Epidermal Growth Factor Receptor OR EGFR OR Receptor, Epidermal Growth Factor OR Tyrosine Kinase Inhibitor OR TKI OR Inhibitors, Tyrosine Kinase OR Targeted Therapy OR Gefitinib OR Erlotinib OR Icotinib OR Afatinib OR Dacomitinib OR Osimertinib):ti,ab |
| #7 | #5 OR #6 |
| #8 | #3 AND #4 AND #7 |

**d:** Search strategy in Web of Science

| # | Query |
| --- | --- |
| #1 | TS=(" Non Small Cell Lung Cancer " OR "NSCLC") |
| #2 | TS=("Brain Metastases" OR "Brain Metastase" OR "Brain Metastasis") |
| #3 | TS=("Radiotherapy" OR "Radiotherapies" OR "Radiation Therapy" OR "Radiation Therapies" OR "Therapy, Radiation" OR "Therapies, Radiation" OR "Radiation Treatment" OR "Treatment, Radiation" OR "Targeted Radiotherapy" OR "Radiotherapy, Targeted" OR "Targeted Radiation Therapy" OR "Therapy, Targeted Radiation" OR "Radiation Therapy, Targeted" OR "Epidermal Growth Factor Receptor" OR "EGFR" OR "Receptor, Epidermal Growth Factor" OR "Tyrosine Kinase Inhibitor" OR "TKI" OR "Inhibitors, Tyrosine Kinase" OR "Targeted Therapy" OR "Gefitinib" OR "Erlotinib" OR "Icotinib" OR "Afatinib" OR "Dacomitinib" OR "Osimertinib") |
| #4 | #1 AND #2 AND #3 |

**Table** **S3** Quality assessment of retrospective studies using the Newcastle-Ottawa scale

| First author/year | Selection | | | |  | Comparability | |  | Outcome | | | Score |
| --- | --- | --- | --- | --- | --- | --- | --- | --- | --- | --- | --- | --- |
|  | Item 1 | Item 2 | Item 3 | Item 4 |  | Item 5 | Item 6 |  | Item 7 | Item 8 | Item 9 |  |
| Byeon/2016[9] | – | * | * | * |  | * | – |  | * | * | – | 6 |
| Jiang/2016[10] | * | * | * | * |  | * | * |  | * | – | – | 7 |
| Chen YS/2016[11] | * | * | * | * |  | * | – |  | * | * | – | 7 |
| Zhu/2017[12] | – | * | * | * |  | * | – |  | * | * | – | 6 |
| Liu YM/2017[13] | – | * | * | * |  | * | – |  | * | * | – | 6 |
| Fan/2017[14] | – | * | * | * |  | * | – |  | * | * | – | 6 |
| Magnuson/2017[15] | * | * | * | * |  | * | * |  | * | * | – | 8 |
| Wang W/2018[16] | – | * | * | * |  | * | – |  | * | * | – | 6 |
| Sung/2018[17] | – | * | * | * |  | * | – |  | * | * | – | 6 |
| Li/2018[18] | – | * | * | * |  | * | – |  | * | * | – | 6 |
| Chen H/2018[19] | * | * | * | * |  | * | – |  | * | – | – | 6 |
| Ke/2018[20] | – | * | * | * |  | * | – |  | * | * | – | 6 |
| Yu/2018[21] | * | * | * | * |  | * | – |  | * | – | – | 6 |
| Wang C/2019[22] | – | * | * | * |  | * | – |  | * | * | – | 6 |
| LEE/2019[23] | * | * | * | * |  | * | * |  | * | – | – | 7 |
| Chen YX/2019[24] | * | * | * | * |  | * | – |  | * | – | – | 6 |
| An/2019[25] | – | * | * | * |  | * | – |  | * | * | – | 6 |
| Saida/2019[26] | – | * | * | * |  | * | – |  | * | * | – | 6 |
| Saruwatari/2019[27] | * | * | * | * |  | * | – |  | * | * | – | 7 |
| He/2019[28] | – | * | * | * |  | * | – |  | * | * | – | 6 |
| Hyun/2020[29] | – | * | * | * |  | * | * |  | * | * | – | 7 |
| Liu YT/2021[30] | – | * | * | * |  | * | – |  | * | * | – | 6 |
| Gu/2021[31] | – | * | * | * |  | * | * |  | * | * | – | 7 |
| Zhao/2022[32] | – | * | * | * |  | * | * |  | * | * | – | 7 |

–, zero point; *, one point. Item 1, representativeness of the exposed cohort; item 2, selection of the non-exposed cohort; item 3, ascertainment of exposure; item 4, demonstration that outcome of interest was not present at start of study; item 5, comparability of cohorts on the basis of the design (study controls for the most important factor); item 6, comparability of cohorts on the basis of the design (study controls for other additional factor); item 7, assessment of outcome; item 8, follow-up long enough for outcomes to occur; item 9, adequacy of follow-up of cohorts.

**Table S4** GRADE assessment

| Outcome | Design | No. of studies /participants | Risk of bias | Inconsistency | Indirectness | Imprecision | Publication bias | Large effect size | Dose effect | GRADE of evidence |
| --- | --- | --- | --- | --- | --- | --- | --- | --- | --- | --- |
| **OS** | Retrospective | 23/3142 | Not serious | Not serious | Not serious | Not serious | Not serious | Not present | Not present | Moderate |
| **iPFS** | Retrospective | 20/2162 | Not serious | Serious (I^2^=62%) | Not serious | Not serious | Serious (Begg's and Egger's test, P<0.05) | Not present | Not present | Very low |
| **iORR** | Retrospective | 11/1295 | Not serious | Not serious | Not serious | Not serious | Not serious | Not present | Not present | Moderate |
| **iDCR** | Retrospective | 9/1006 | Not serious | Not serious | Not serious | Not serious | Not tested (n < 10) | Not present | Not present | Moderate |
| **Subgroup analyses for OS** | | | | | | | | | | |
| Asymptomatic BMs | Retrospective | 3/320 | Not serious | Not serious | Not serious | Serious (only 3 studies) | Not tested (n < 10) | Not present | Not present | Low |
| Exon 19 mutation | Retrospective | 2/152 | Not serious | Not serious | Not serious | Serious (only 2 studies) | Not tested (n < 10) | Not present | Not present | Low |
| Exon 21 mutation | Retrospective | 2/199 | Not serious | Serious (I^2^=83%) | Not serious | Serious (only 2 studies) | Not tested (n < 10) | Not present | Not present | Very low |
| 1-3 BMs | Retrospective | 2/189 | Not serious | Not serious | Not serious | Serious (only 2 studies) | Not tested (n < 10) | Not present | Not present | Low |
| >3 BMs | Retrospective | 2/161 | Not serious | Not serious | Not serious | Serious (only 2 studies) | Not tested (n < 10) | Not present | Not present | Low |
| Male | Retrospective | 2/170 | Not serious | Not serious | Not serious | Serious (only 2 studies) | Not tested (n < 10) | Not present | Not present | Low |
| Female | Retrospective | 2/209 | Not serious | Not serious | Not serious | Serious (only 2 studies) | Not tested (n < 10) | Not present | Not present | Low |
| **Subgroup analyses for iPFS** | | | | | | | | | | |
| Asymptomatic BMs | Retrospective | 5/427 | Not serious | Not serious | Not serious | Not serious | Not tested (n < 10) | Not present | Not present | Moderate |
| Symptomatic BMs | Retrospective | 4/253 | Not serious | Serious (I^2^=87%) | Not serious | Serious (sample size ＜300) | Not tested (n < 10) | Not present | Not present | Very low |
| Exon 19 mutation | Retrospective | 4/283 | Not serious | Serious (I^2^=61%) | Not serious | Serious (sample size ＜300) | Not tested (n < 10) | Not present | Not present | Very low |
| Exon 21 mutation | Retrospective | 4/318 | Not serious | Serious (I^2^=65%) | Not serious | Not serious | Not tested (n < 10) | Not present | Not present | Low |
| 1-3 BMs | Retrospective | 4/283 | Not serious | Not serious | Not serious | Serious (sample size ＜300) | Not tested (n < 10) | Not present | Not present | Low |
| >3 BMs | Retrospective | 5/423 | Not serious | Not serious | Not serious | Not serious | Not tested (n < 10) | Not present | Not present | Moderate |
| Male | Retrospective | 4/267 | Not serious | Not serious | Not serious | Serious (sample size ＜300) | Not tested (n < 10) | Not present | Not present | Low |
| Female | Retrospective | 4/367 | Not serious | Not serious | Not serious | Not serious | Not tested (n < 10) | Not present | Not present | Moderate |

Abbreviations: GRADE, Grading of Recommendations, Assessment, Development and Evaluations; OS, overall survival; iPFS, intracranial progression-free survival; iORR, intracranial objective response rate; iDCR, intracranial disease control rate; BMs, brain metastases.


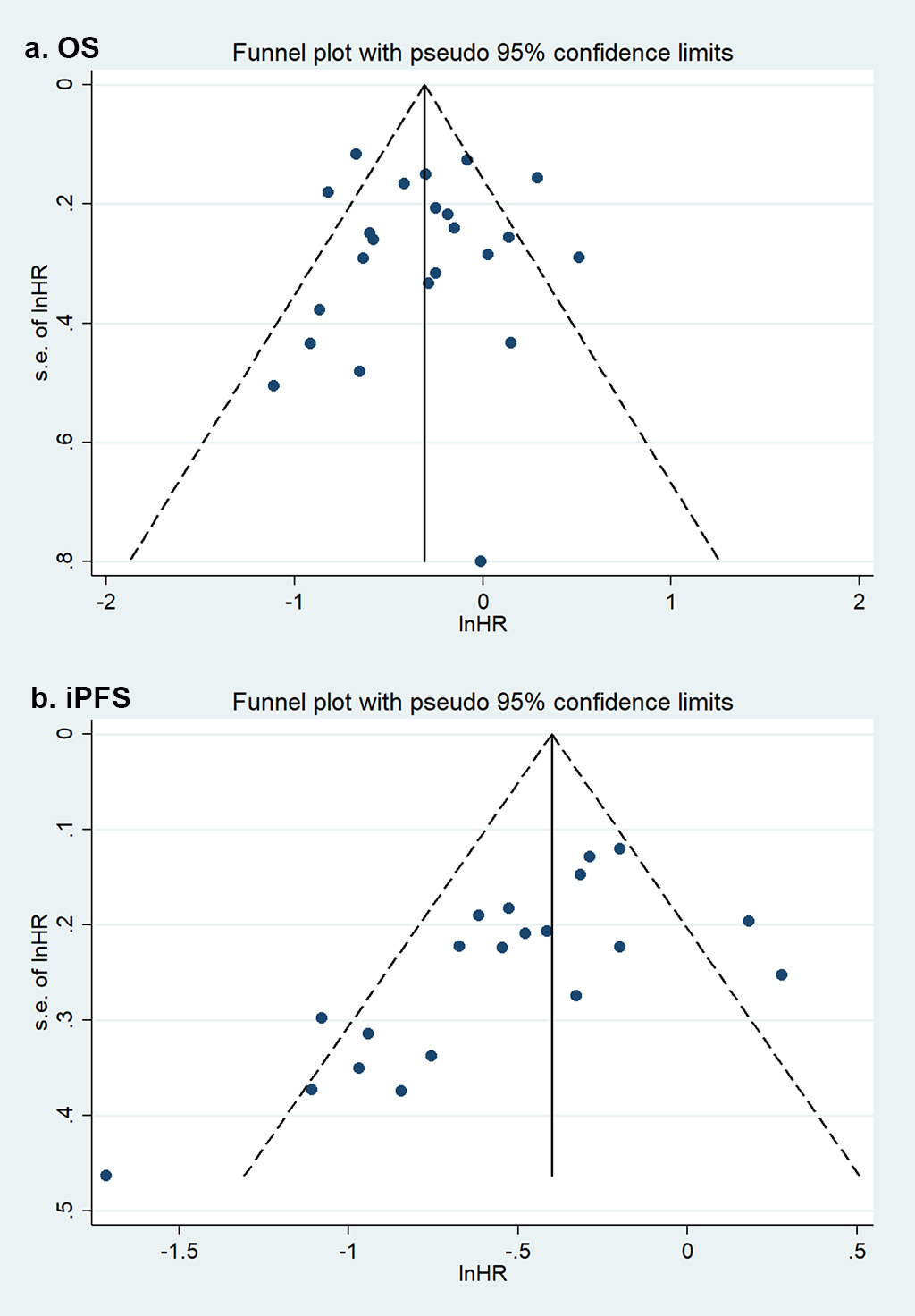


**Fig** **S1** Funnel plots of publication bias. OS, overall survival; iPFS, intracranial progression-free survival.


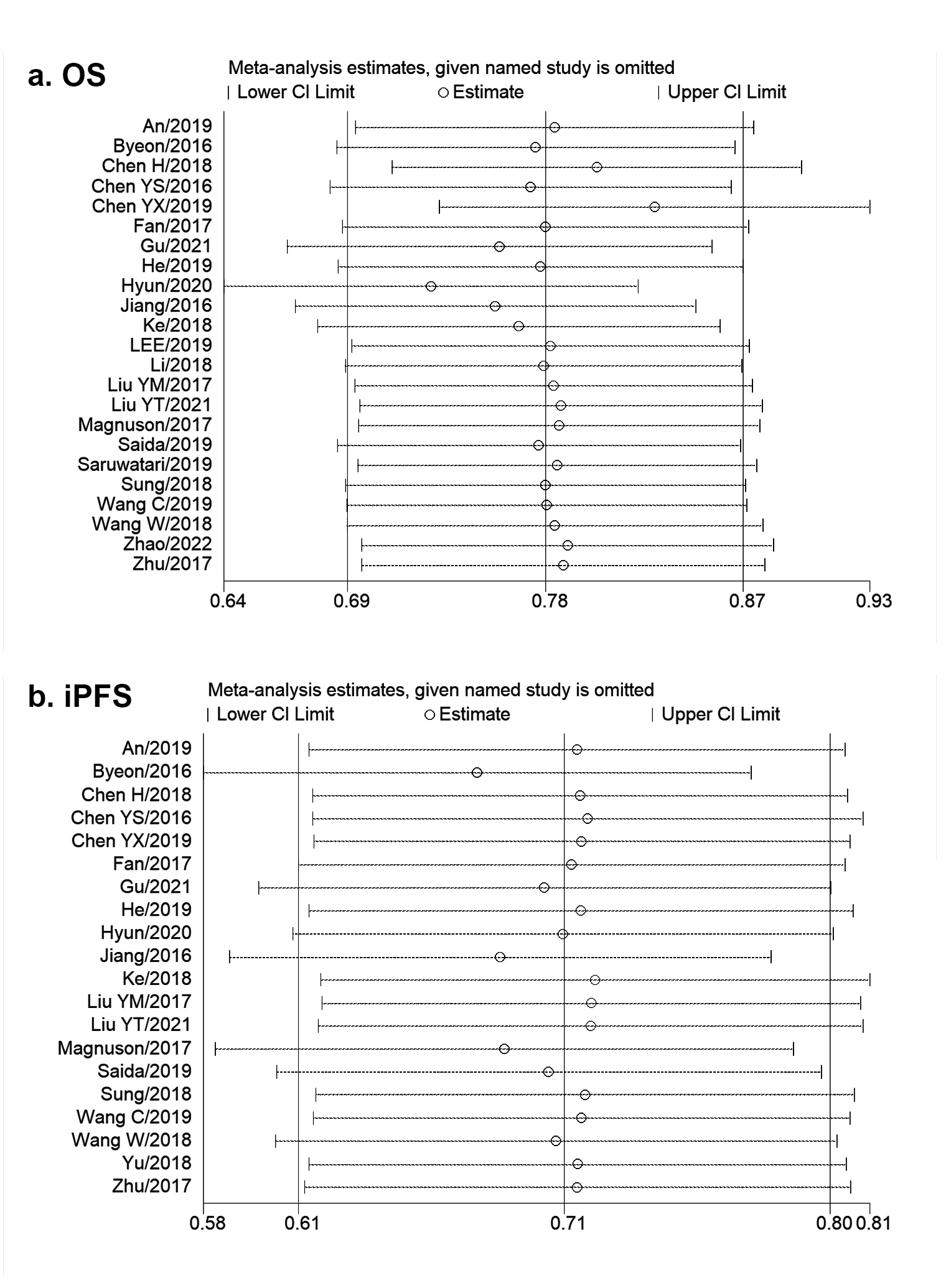


**Fig S2** Sensitivity analysis. OS, overall survival; iPFS, intracranial progression-free survival.
